# Current Advances in Robotics for Head and Neck Surgery—A Systematic Review

**DOI:** 10.3390/cancers13061398

**Published:** 2021-03-19

**Authors:** Felix Boehm, Rene Graesslin, Marie-Nicole Theodoraki, Leon Schild, Jens Greve, Thomas K. Hoffmann, Patrick J. Schuler

**Affiliations:** 1 Department of Otorhinolaryngology, Head and Neck Surgery, Ulm University Medical Center, 89075 Ulm, Germany; rene.graesslin@uniklinik-ulm.de (R.G.); marie-nicole.theodoraki@uniklinik-ulm.de (M.-N.T.); leon.schild@uni-ulm.de (L.S.); jens.greve@uniklinik-ulm.de (J.G.); ent.department@uniklinik-ulm.de (T.K.H.); 2 Surgical Oncology Ulm, i2SOUL Consortium, 89075 Ulm, Germany

**Keywords:** robotics, robotic surgical procedures, TORS, head and neck neoplasms, haptics, costs

## Abstract

**Simple Summary:**

The means of therapy in oncologic diseases have been developing continuously over the past years, intending to improve the overall survival and quality of life of affected patients. In head and neck oncology the surgical therapy is one of the key pillars in curative treatment. The standardized surgical techniques are supplemented and improved by the application of technical devices. The ambition is the reduction in peri- and postoperative morbidity, hospitalization time, and the enhancement of functional outcome. In other surgical specialties, the application of robotics is widely seen as standard. The purpose of this review is to outline the current status of robotics in head and neck surgery in the context of the present literature, to demonstrate reasonable application fields, and to discuss the expenditure of the usage of such tools. Furthermore, this review offers an overview of current research in this field.

**Abstract:**

**Background.** In the past few years, surgical robots have recently entered the medical field, particularly in urology, gynecology, and general surgery. However, the clinical effectiveness and safety of robot-assisted surgery (RAS) in the field of head and neck surgery has not been clearly established. In this review, we evaluate to what extent RAS can potentially be applied in head and neck surgery, in which fields it is already daily routine and what advantages can be seen in comparison to conventional surgery. **Data sources.** For this purpose, we conducted a systematic review of trials published between 2000 and 2021, as well as currently ongoing trials registered in clinicaltrials.gov. The results were structured according to anatomical regions, for the topics “Costs,” “current clinical trials,” and “robotic research” we added separate sections for the sake of clarity. **Results.** Our findings show a lack of large-scale systematic randomized trials on the use of robots in head and neck surgery. Most studies include small case series or lack a control arm which enables a comparison with established standard procedures. **Conclusion.** The question of financial reimbursement is still not answered and the systems on the market still require some specific improvements for the use in head and neck surgery.

## 1. Introduction

In a variety of surgical procedures, especially in the field of visceral surgery, gynecology, or urology, robot-assisted surgery (RAS) is seen as an undisputed standard. Therefore, it is not surprising that possible applications in the field of head and neck surgery are progressively explored and studies for clinical testing of the various technical systems, which are not yet used nationwide, are initiated.

The autonomous conduction of a surgical procedure through a robotic device is currently inconceivable in medical applications, because of concerns about patient safety and technical complexity. Still, current RAS systems are advanced, computerized operation tools aiming at an improvement of surgical access and visualization in anatomical regions that are difficult to reach. They further aim to enhance the precision of the surgeon, e.g., through software-controlled reduction in natural tremor or through special surgical instruments, which allow for greater flexibility and more degrees of freedom than the human hand. In general, the reduction in surgical trauma, improvement in the surgical outcome and possibly even a shorter hospitalization time are discussed as potential advantages of RAS [1,2]. This review evaluates to what extent the abovementioned possible theoretical benefits of RAS can be applied advantageously in head and neck surgery for the patients as well as the surgeons.

For this purpose, we describe the application and potential of RAS systems in head and neck surgery structured by anatomical regions. Eventually, we aim to answer the question, how current RAS systems perform in comparison to conventional surgical methods in surgical access morbidity, reduction in the operation time, and patient outcome [3,4]. Furthermore, we offer an overview of the currently available and most used systems on the market. In the end, the application of any type of robot-assisted surgery is accompanied by an increased financial effort, that has to be discussed. Additionally, we present an overview of the current study landscape and a perspective concerning future robotic research in the medical sector worldwide.

**Figure 1:**
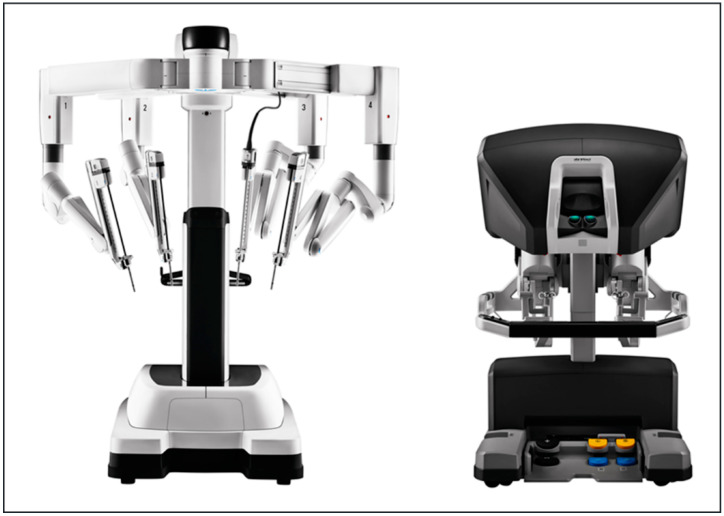
DaVinci Xi^®^. Multi-port robotic system with camera arm and several working arms for the use of different surgical instruments. Reproduced with kind permission from Intuitive Surgical^®^, Inc., Sunnyvale, CA, USA, © 2021.

Currently, the global market leader for robot-assisted surgery is Intuitive Surgical (Sunnyvale, CA, USA), which distributes the world-wide known DaVinci system (Figure 1).

However, the surgical instruments were initially developed for abdominal surgery, and the wide dimensions of the system do not seem to fit for the narrow anatomic regions in head and neck surgery. Therefore, other companies aim to introduce their systems into the market, which are specially developed to fit the requirements in the narrow head and neck anatomy [5].

In Anglo-American countries, RAS is widely used in clinical routine for the surgical treatment of benign and malignant tumors of the oropharynx. Surgeons in Asian regions apply these systems routinely in transaxillary surgery of the thyroid gland aiming at the avoidance of visible scars in aesthetically relevant skin regions as the neck and the face. Here, the avoidance of visible scars is seen as a large advantage compared to conventional surgical techniques.

Available RAS systems differ primarily in the characteristics of control and sensor technology. The system control includes settings for power regulation, remote control, as well as position and speed regulation of the surgical instruments. Sensor technology refers to the flow of information from the surgical system to the surgeon and is composed of intraoperative imaging, imaging-supported navigation, and haptics. In general, active telesurgery systems (e.g., DaVinci, Flex, MicroRALP) can be differentiated from passive robot assistance systems (e.g., SOLOASSIST, Cirq). Latter supports the surgeon in the visualization of the surgical field, the haptic, and the navigation of surgical instruments [6].

## 2. Methods

Criteria for considering studies for this review were trials including at least more than 2 participants. Participants were patients with resectable diseases of the head and neck region. The surgical intervention had to be performed with robot-assisted surgery or another technological device facilitating the surgical handicraft. Types of outcome measures included intraoperative complications including injury of blood vessels and nerves, postoperative complications including hemorrhage, thrombosis, and wound infection as well as mortality, postoperative morbidity, overall survival, disease-free survival, operating time, instrument setup time, duration of hospital stay, postoperative quality of life, and cost assessment of the surgical procedure.

We conducted structured literature research in the databases of PubMed for published trials and clinicaltrials.gov for current ongoing studies. We used the keywords “robot-assisted surgery,” “head and neck,” “transoral robotic surgery,” and “TORS.” All studies written in English and German published before 2000 were considered, while publications that describe only single case-reports were excluded. Publication status had to be printed or e-pub ahead of print. For current clinical trials, only studies that started after 2000 were considered, and abandoned trials were excluded.

Furthermore, we hand-searched different international government initiatives for fundamental robotic research worldwide. We included only projects that could potentially be used in the medical field of robotics in the future. Projects aiming rather at developing industrial robots or intended for the end consumer were excluded.

**Figure 2:**
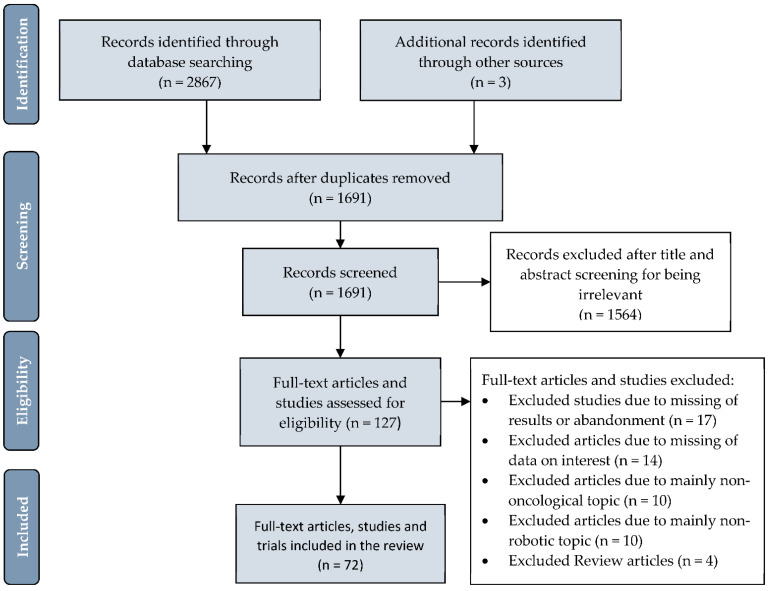
PRISMA (Preferred Reporting Items for Systematic Reviews and Meta-Analyses) flowchart depicting the number of identified articles and trials, those screened and final number included in the systematic review.

For the sake of clarity, we structured our research results by anatomical regions for published studies and presented the topics “costs,” “current clinical trials,” and “government-funded robotic research” in separate sections. Figure 2 depicts the PRISMA flowchart demonstrating the process of article retrieval and screening.

## 3. Results

### 3.1. Pharynx

In selected tumor centers, oropharynx-tumors in early tumor stages (T1-2) have been routinely treated by transoral robot-assisted surgery (TORS) for several years [7]. Retrospective analysis of patients treated this way shows good results concerning organ function, quality of life, as well as survival rates. Unfortunately, clinical trials in this particular field rarely describe the specific treatment protocols that TORS is compared to. Various studies show a good quality of life for TORS patients [8,9].

A retrospective study by Park et al. compares TORS to open transcervical surgery in patients with T1-4 tumors of the hypopharynx. There was no difference in overall survival after 5 years between the two patient groups. The patients treated with TORS showed a shorter postoperative recovery time, a shorter duration of hospital stay as well as better postoperative swallowing function [10]. Another retrospective study points to longer disease-free survival of patients treated with TORS in comparison to transoral laser microsurgery (TLM) [11]. For further evaluation of TORS, randomized prospective trials that compare TORS especially with transoral laser microsurgery or conventional open would be desirable.

Similarly, the parapharyngeal space increasingly draws the interest of the TORS surgeons [12,13]. A case series outlines the removal of parapharyngeal tumors through transoral access to the surgical site using TORS. In this study, it was possible to remove tumors up to a maximum size of 5 cm × 6 cm × 7 cm [14]. After pharyngeal tumor excision, studies describe the successful use of TORS for tissue reconstruction with free flaps including the microvascular anastomosis sutures to the neck blood vessels [15]. Furthermore, known innovative tissue reconstruction techniques may benefit from the application of TORS. One study describes the TORS-based reconstruction of a tissue defect after lateral oropharynx resection and radical tumor tonsillectomy with a naso-septal flap being pulled through a transpalatal tunnel [16].

Besides its application, in oncologic surgery, TORS is regularly used in the treatment of obstructive sleep apnea syndrome (OSAS). For instance, the application is described for the performance of a uvulopalatopharyngoplasty. However, even more complex surgical interventions like the resection or suspension of the tongue base, plastic surgery for mandibular protrusion, or a supraglottoplasty are presented in the current literature [17,18]. Nevertheless, the application of TORS in clinical routine for OSAS is currently controversial especially concerning the high financial effort.

**Figure 3:**
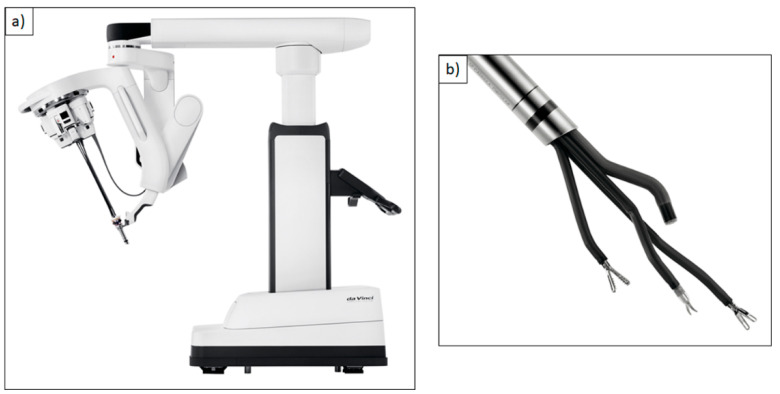
(**a**) DaVinci Single-Port (SP)**^®^** robotic system; (**b**) Single 2.5 cm cannula containing three instrument arms and an endoscope. Reproduced with kind permission from Intuitive Surgical^®^, Inc., Sunnyvale, CA, USA, © 2021.

The Flex System (Medrobotics, Raynham, MA, USA) is approved for application in transoral head and neck surgery since 2016 [5,19]. It is a computer-controlled flexible endoscope system, which adapts to the patient’s anatomy upon the transoral entering of the pharynx. This means that head reclination is not necessary, which is especially useful for patients with unfavorable anatomic preconditions, and therefore the Flex System can be an alternative to transoral laser microsurgery. Possible limitations like restricted cervical spine reclination or limited mouth opening can be overcome through the Flex System [5,20]. The financial effort in comparison to the DaVinci system is considerably smaller, but still enormous compared to the established laser microsurgery. So far, there are no clinical trials that compare the Flex System and the LMS concerning visualization of the glottis area or duration of the surgical procedure. The DaVinci Single-Port (SP) is the latest generation of the DaVinci robot and is so far still in clinical testing (Figure 3a,b).

Several clinical trials describe successful procedures on a total of 88 patients with oropharynx cancers [21,22]. Advantages in comparison to the preceding model can be seen in improved visualization and a revised surgical instrument handling.

**Figure 4:**
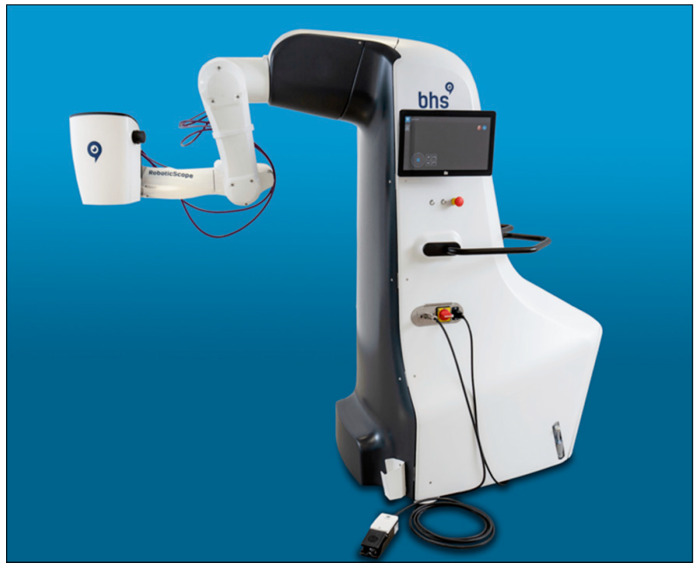
RoboticScope^®^-system with a high-resolution 3D-camera and a head-mounted display. Reproduced with kind permission from BHS Technologies^®^, Innsbruck, Austria, © 2020.

Other potential robotic systems like Senhance^®^ (Asensus Surgical), Enos^®^ (Titan Medical), or the Versius^®^ (CMR Surgical) are either still in preclinical testing or have been applied in abdominal surgery only so far [23,24]. Another interesting development to consider are exoscopes. They consist of high-resolution cameras, which acquire images of the operation site that can be transferred to an external display. Exoscopes are a contrast to conventional surgical microscopes because they allow for a change of the surgeon’s body position without disconnecting the view of the surgical field. Depending on model and provider, they can include many more functions. One example of such an exoscope system is the RoboticScope of the company BHS Technologies (Figure 4).

The device consists of a high-resolution 3D-camera, which is mounted on a microscope holding arm. Visualization is carried out through a virtual reality headset, which also allows for the steering of the camera using head movements. It could be a useful tool in oncologic microsurgery. Especially performing the micro-anastomosis of free-flap transplants during reconstruction after extended tumor resection could be a possible field of application. A similar system is the VITOM 3D HD. It is equipped with a manual or alternatively with a motorized holding arm. The control of the system is possible manually or through a 3D computer mouse, not via head movements like is the case with the RoboticScope [25]. Exsoscopes can also be combined with other modern imaging modalities, e.g., multispectral imaging. The ORBEYE^®^ exoscope (Olympus, Tokyo, Japan) contains besides the conventional white light imaging, which is available in 3D with 4K-resolution, also fluorescence imaging modes. The system offers the surgeon the possibility to choose between blue light imaging or an infrared mode, which enables a very good tissue contrast after application of ICG (indocyanine green). Furthermore, modern narrow-band imaging (NBI) applies to the system as well. NBI describes hemodynamic-like imaging from the basis of hemoglobin autofluorescence [26].

### 3.2. Larynx

In Europe, benign and malignant neoplasms of the larynx are usually treated with transoral microsurgical procedures and, if necessary, with the additional use of laser technique. In large tumors, the open transcervical approach through laryngectomy or partial resection of the larynx is a surgical standard. Several working groups describe surgical laryngectomy through transoral access using TORS [27,28,29,30]. However, the groups merely describe the technical feasibility of the procedure. A randomized clinical trial with the evaluation of functional results, overall survival, and quality of life remains to be seen.

Good results could be achieved in the treatment of supraglottic larynx cancers using TORS. A multicentric study analyzed the cases of 122 patients retrospectively after partial larynx resection with TORS [30]. Patients in the tumor stages T1–T3 were successfully treated. The authors describe better visualization of the operation site as well as easier mastering of procedures with TORS in comparison to transoral laser microsurgery. In most cases the performance of a tracheostomy was not necessary, leading to lower postoperative morbidity as well as faster convalescence. For visualization of the operation site and performance of the surgery, most studies use the DaVinci system with the aid of a Feyh-Kastenbauer-retractor. Additionally, supraglottic laryngectomy (LE) has been described with the new DaVinci Single-Port system as well as the Flex System [11,31]. The application of the Flex system with smaller surgical instruments for the larynx in glottic tumors is so far limited to a few cases with heterogeneous success [32,33].

Another group investigated the functional results and the quality of life after TORS-larynx-surgery. They performed supracricoid partial laryngectomy with cricohyoidoepiglottopexy in two patient cases. Quality of life, phonation, and swallowing function were examined. The average values of the two study patients were compared to a patient group consisting of 69 patients, which were treated with an open transcervical surgery between 1983 and 1996. Concerning the swallowing function, no difference between the two groups could be detected. The maximum phonation time, however, was better in the TORS group and comparable to the time of a healthy person [29]. The validity of this study, however, is limited with only two patients in the study group. In conclusion, there are currently no studies that show an advantage of robot-assisted surgery in comparison to the transoral laser microsurgery, which is widely established in Europe. Taking into consideration the considerably lower costs of transoral laser microsurgery in comparison to TORS, a standard application of TORS in clinical routine does not seem to be feasible yet.

The MicroRALP system is a cooperation project, which has been promoted in preclinical testing in the European Union in France, Italy, and Germany [34]. The system consists of a tele-controlled laser instrument that enables a high-precision semiautomatic vocal cord surgery after respective programming. (www.microralp.eu accessed on 31 January 2021). The system is currently under further development by the Leibniz University (Hannover, Germany) [35,36].

**Figure 5:**
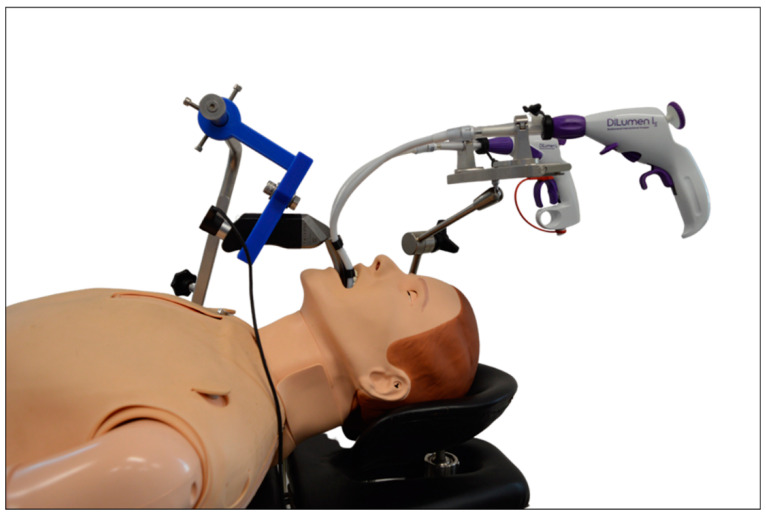
Curved prototype of a video laryngoscope equipped with flexible instruments for laryngeal surgery. Reproduced with kind permission from Schuler et. al., Ulm University Medical Center, Ulm, Germany, © 2020.

Depending on individual anatomic conditions, transoral surgery of the larynx can prove to be challenging especially in patients with limited head reclination or tumor involvement of the anterior commissure. As a solution, Schild et al. propose a curved prototype for laryngeal surgery equipped with flexible instruments (Figure 5).

In the user study (n = 19) with a porcine ex vivo laryngeal model, the participants were able to reach and manipulate important laryngeal landmarks, such as the anterior commissure, in an acceptable time frame. They reported a good general impression and no head and neck strain using the system with a 40-inch external monitor. The results of this user study suggest that the prototype is an interesting and low-priced alternative to the previously available systems in robotic larynx surgery, especially concerning visualization and accessibility of the operation site [37,38].

### 3.3. Paranasal Sinuses and Skull Base

At first impression, RAC exhibits considerable advantages in the surgery of the skull base or the paranasal sinuses in comparison to the endonasal surgery established by Messerklinger in 1978 [39]. Advantages include the possibility of a 3D-HQ visualization, the non-existing necessity of an experienced surgical assistant for endoscope movement, the compensation, and filtering of natural tremor as well as the increased number of degrees of freedom in the movement of surgical instruments [40]. However, considering current TORS systems on the market, it becomes obvious that transoral or transnasal access to the paranasal sinuses or the skull base is not primarily possible. Currently, surgery of the skull base is mainly conducted with extended endoscopic techniques through both nasal cavities in a 4-handed technique [41]. To enable the application of TORS, experimental setups use a transpalatal access to the operation site. Unfortunately, this leads to potential complications like postoperative velopharyngeal insufficiency during swallowing, fistula formation, or functional disorder of the Eustachian tube [4,42,43]. Henry et al. describe the resection of clivus chordomas invading the nasopharynx using TORS in three patients. Surgery was conducted using transpalatal access, in one case supported by an endoscopic view through the nose. The amount of resection was comparable to the established open-access technique while being less invasive [44]. Of note, it was not possible to reach the anterior skull base. Furthermore, there are currently no drilling devices for TORS systems available, which are necessary for vital bone removal.

A combination of TORS with surgical navigation systems, which are mandatory in pre-operated patients or patients with difficult anatomic conditions, is not yet possible [40]. Besides missing haptic feedback, there are currently no suction instruments for clearance of the operation site from blood or secretion. This task still needs to be fulfilled by an experienced surgical assistant. Furthermore, as previously mentioned, a large disadvantage of RAS is the missing of haptic feedback [40]. A binasal access would provide an alternative to the palate split dissection. Currently, there are no RAS systems, which provide adequate mobility for endonasal movement as currently available instruments seem to be too large [40,45]. All in all, RAS attracts wide interest in the field of the skull base and paranasal sinus surgery, which shows especially in a large number of cadaver studies [40].

A combination of classic endoscopic transnasal surgery techniques and TORS for the resection of a nasopharynx carcinoma via soft palate splitting and bone resection was first used in clinical application in 2012. No postoperative complications could be detected [43]. Carrau et. al. described the resection of two aggressive tumors of the skull base through a combination of RAS and conventional surgery. One case was a clivus chordoma, the other patient presented with an adenoid-cystic carcinoma. In one patient case, a mild velopharyngeal insufficiency occurred postoperatively after split plate dissection [4]. A larger study examined 12 patients after salvage nasopharyngectomy through soft palate splitting with RAS. One patient developed a postoperative fistula on the transition between the soft palate and hard palate. This was successfully treated using an obturator plate. The results showed a high 2-year local tumor control rate of 86%. The 2-year overall survival and disease-free survival were 83% and 61%. The operating time was comparable to open surgery [46].

**Figure 6:**
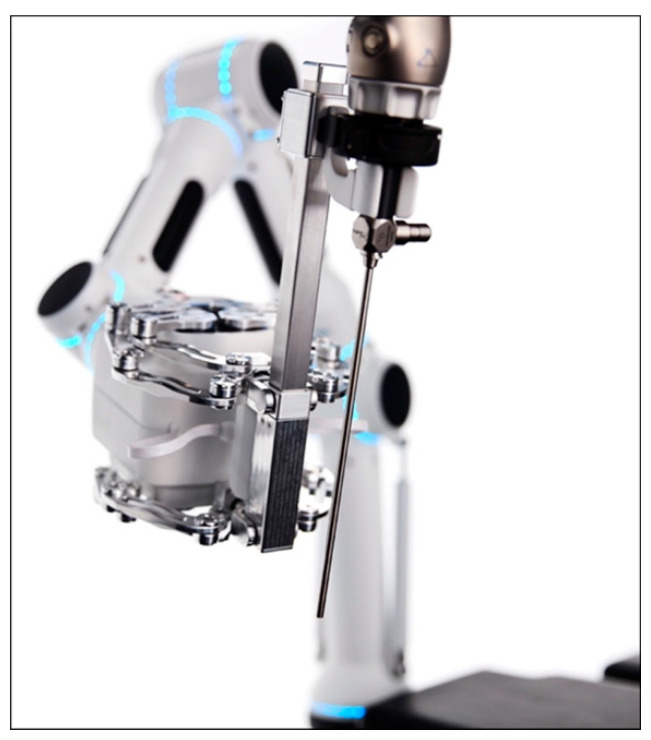
Cirq^®^. Robot-assisted endoscope guidance system with a mechatronic arm consisting of several segments with 7 degrees of freedom and the possibility to attach a conventional endoscope. Reproduced with kind permission from Medineering/Brainlab^®^, Munich, Germany, © 2021.

Especially flexible systems should be further developed to allow possibly transoral access of the nasopharynx or the skull base without invasive palate splitting. Systems suitable for this application could be the flex-system of the company Medrobotics or a continuum robot [40,47]. A computer-controlled endoscope holding system called Cirq (Medineering/Brainlab, Munich, Germany) acquired approval for clinical application in 2017 (Figure 6). Since then, it could be shown that this system is feasible for surgery of the nasolacrimal duct. Commercial availability is currently pending [48].

### 3.4. Thyroid Gland and Neck Dissection

Traditional open approaches in thyroid surgery and neck dissection are always accompanied by visible scaring. In some cultures, especially in countries of South East Asia, and in some professions, e.g., in model business, special attention is given to aesthetic appearance. In 2005, a solitary lymph node was removed for the first time through a transaxillary access using RAS. Meanwhile, good results have been shown on large patient cohorts [1,49,50].

Still, the importance of aesthetics remains ethically questionable compared to the safest oncologic resection of malignant tumors of the neck. Due to better visualization of the operation site, it is our strong belief that the transcervical approach is to be favored at present despite various advancements in robot-assisted surgery [51,52].

For robot-assisted neck dissection, which was firstly described by Kang et al. in 2010, a retroauricular access, as well as axillary access to the operation site, is described [53]. Two studies show identical rates of complications as well as a comparable number of removed lymph nodes for RAS compared to open surgery. Lee et al. pay particular attention to complications regarding the accessory nerve. Permanent damage of the nerve could be detected neither in the RAS-group (10 patients) nor in the conventionally operated control group (16 patients) [54,55]. In the context of the low number of studies and the missing data to overall-survival or disease-free-survival, the study results are only reliable to a limited degree. In European culture, the aesthetic results and the avoidance of visible scarring is of limited importance. This is why a routine application of RAS in thyroid surgery or neck dissection is not expected soon.

One possible field of application for RAS is the resection of retropharyngeal lymph nodes. Two studies describe TORS for this indication in patients with tonsil cancer. In the case of assumed retropharyngeal metastases, radiation therapy is often the choice of treatment even with missing histologic confirmation due to the high morbidity of conventional transcervical or transmandibular surgical access.

Based on imaging alone, the decision for or against adjuvant therapy is often difficult. A retropharyngeal lymph node resection provides the advantage of histologic confirmation of metastases and thus avoids unnecessary adjuvant treatment or mistakenly omitted therapy. In both studies, TORS was used for oropharyngectomy, afterward, TORS-supported lymph dissection of the retropharyngeal area followed with access through the primary resection site. Additionally, conventional neck dissection was performed. In a study of Park et al. with 3 patients, no postoperative wound infections, bleedings, or other serious complications could be detected. The TORS procedure is described as feasible with morbidity as lower in comparison to conventional surgery [56]. A study by Troob et al. compared 37 patients with oropharyngeal cancers and conventional neck dissections to patients with additional retropharyngeal lymph node dissections with TORS (30 patients). There were no differences in duration of hospital stay, dwell time of feeding tube, swallowing function, postoperative complications, or bleedings [57]. A comparison between the oncologic outcome and the surgery times has been omitted in the study.

A distinct advantage of TORS in retropharyngeal lymph node dissection is currently not evident, at least in patients with extensive oropharyngectomy, as the partial removal of the pharynx already enables access to the retropharyngeal space.

The following table (Table 1) summarizes the different robot systems mentioned in the preceding chapters with a focus on their distinct advantages and disadvantage and their field of use in the ENT region.

### 3.5. Costs

In established surgical procedures of the head and neck, the majority of the expenses are staff costs, whereas in robotic-assisted surgery, the acquisition and material expenses of robotic technology present as the key costs. This is among others attributable to the monopoly position of the system-producers in the providing of surgical instruments [58]. Account analysis of Dombree et al. shows that expenses for oncologic laryngectomy applying the DaVinci system are up to 90% higher than those of the established surgical procedure [59]. Most studies available compare TORS to standard chemoradiotherapy. TORS proves to be cost-effective in comparison to chemoradiotherapy if patients can be treated solely with surgery without any adjuvant (chemo)radiotherapy. The majority of this group are patients with small, easily accessible tumors in less advanced tumor stages without metastases [60,61]. In these cases, the application of TORS is questionable, and thus the significance of these studies is limited. Analyses that compare explicitly TORS to established surgical procedures would therefore be desirable.

Primarily invisible cost factors must not be forgotten. Benito et al., e.g., describe a cost reduction in the application of TORS attributable to the employment of a specific teeth protector. The costs for teeth damage that in some countries has to be paid for by the treating clinic could be significantly reduced by this device [62]. Other cost benefits of TORS could be seen in the avoidance of complications through a reduction in surgical invasiveness, e.g., the necessity of tracheostomy or feeding tubes. Furthermore, several studies describe a reduction in hospitalization time for the use of RAS [63]. According to our expertise, the additional costs for a surgical procedure assisted by a DaVinci system are approximately 7100 USD/6000 EURO. These costs consist of maintenance charges, acquisition costs, as well as instrument reprocessing and acquisition. Currently, in most countries, there is no distinct reimbursement of costs for new treatment approaches like robot-assisted surgery [64]. Consequently, RAS cannot be performed economically viable.

### 3.6. Clinical Trials

For an objective assessment of robot-assisted surgery in the field of head and neck surgery, clinical trials are of utmost importance. Therefore, the extremely low amount of prospective randomized clinical trials is striking.

Lee et al. compared robot-assisted neck dissection to established surgical techniques. Both approaches showed similar results concerning the number of removed lymph nodes (NCT01488669) [54]. Another not-randomized study compares the safety and feasibility of TORS in salvage surgery of tumors of the oropharynx (NCT00473564). The study group (n = 7) who obtained treatment with TORS exhibited a lower amount of persisting feeding tubes and tracheostomies after 6 months when compared to the control group with the established surgical technique (n = 14). There were no postoperative complications in the RAS group [65].

An ongoing trial of Lin et al. is particularly interesting. The aim of the study is a randomized comparison of RAS to standard surgery (transoral or open transcervical) for benign or malignant tumors of the pharynx and larynx. Recruitment of the study has already been completed, but results have not been published so far (NCT00918762). In another randomized trial, Palma et al. compare the treatment of patients with squamous cell carcinoma of the oropharynx with TORS in combination with established standard neck dissection to primary chemoradiotherapy (NCT01590355). The patient recruitment is completed with a target number of 68 patients. Currently, follow-up examinations are still ongoing.

A large-scale study by Ozer et al. aims at treating 360 patients with benign and malignant tumors of the oropharynx and larynx with TORS. The study is still recruiting. One point of criticism remains the lack of a control group in the study design (NCT01473784).

A multicentric, non-randomized trial examined the safety of the Flex System (Medrobotics). The visualization and surgical removal of the target lesion were possible in 95% of the treated patients. There were no serious or unexpected complications linked to the usage of the Flex system [66].

The lack of randomized trials comparing TORS to standard treatment is generally considered problematic. There are indeed some interesting studies, e.g., by Gross et al., who compare TORS to proton irradiation in patients with low-risk oropharyngeal cancer (NCT02663583). Still, this study compares two treatments, and neither can be considered standard therapy. This is why, the transferability to clinical routine is questionable. The ORATOR-study (NCT01590355), which includes 68 patients with oropharynx carcinomas (T1/2), is a randomized study and compares primary chemoradiotherapy to TORS + neck dissection [67]. After a follow-up time of 2 years, the trial showed a better dysphagia score in favor of the primary chemoradiotherapy [68]. However, in the surgical study group, the following points that affect swallowing function negatively should be taken into consideration: (I) standard tracheostomy was performed even in small primary tumors (T1/2), and (II) an extraordinary large safety margin of 1 cm was used.

### 3.7. Robotic Research

Concerning the variety of interesting research approaches, the following presents a general overview of robot-based projects. Table 2 shows different governmental initiatives for general robotic research and development worldwide. Table 3 shows selected international research projects with a focus on robotics.

The South East Asian countries, the United States of America, and the European Union are amongst the leading countries in robotic research and development [69].

China, e.g., began research on industrial robots in 1972. Since then, many development and research projects in the field of robotics have been introduced. In 2016, the latest government-funded project the “Robot Industry Development Plan” (2016–2020) was announced. Besides industrial robots, welding robots, etc., one of the main focus points of the initiative was surgical robots and intelligent nursing robots. The goal of the project is to strengthen technological innovation capacity and to expand the industrial scale of robotics in China. New-type mechanisms, sensing, control, and bionics are developed for human-machine natural interaction and collaboration. Another Chinese research program is the “Key Special Program on Intelligent Robots” of 2019. The initiative contains 33 planned projects in the field of robotics including the development of micro-nano field-control robots for targeting drug delivery to human cells and the development of robotic systems for close-range radiotherapy or minimal invasive surgery like the minimal invasive implantation of an artificial cochlea.

In 2015, Japan announced the “New Robot Strategy” with an annual budget of 351 million dollars in 2019. The initiative focuses mainly on 5 sectors, one of them being nursing and medical robotics. Key targets are simplification of inspection and unification of diagnosis, treatment, and prevention [69].

In 2018, the United States announced the governmental funded “National Robotics Initiative 2.0: Ubiquitous Collaborative Robot (NRI-2.0).” The project promotes human assistive robotic devices that serve as smart personal protective equipment and the integration of robotic arms and hands [69].

Two major robotic research projects are funded by Korea. The “Implementation Plan for Intelligent Robots” of 2018 aims, e.g., to develop a single-port surgical robot with flexible joints for oral or laparoscopic surgery and to facilitate the investment expansion in robot fields. “The 3rd Basic Plan on Intelligent Robots” was announced in 2019 up to 2023. Development of the robot industry as a core industry and support of innovation in manufacturing and services is one main goal. The program includes the development of operation robots as well as rehabilitation robots [69].

In the “Horizon 2020” initiative, the European Union promotes research and innovation in its member countries. The duration is set from 2014 to 2020. The aim of the initiative is a knowledge-based society and a competitive economy in Europe. “Horizon 2020” supports several robot-assisted research programs. One project that engages in surgical robotics is called “Smart Wearable Robotic Teleoperated Surgery” (SMARTsurg; project number 732515). The project directors develop a surgical robot with a master-slave arrangement, which shows some similarities to the popular DaVinci robot. However, visualization of the surgical field is ensured by augmented reality 3D-glasses and the instrument steering includes a haptic feedback option [70].

Since 2017, the German research foundation (DFG) promotes a post-graduate program about “Soft Tissue Robotics” (GRK 2198). Doctorates of the program engage in fundamental research on the development of robots that can interact with soft tissue. The need for this kind of robotic assistant is seen especially in the automobile industry, agricultural economics, and medicine. The post-graduate program is based on international cooperation with Auckland, New Zealand.

A similar topic promoted by the priority program of the DFG since 2019 is called “Soft Material Robotic Systems” (SPP2100) and is coordinated in Hannover (Germany). In contrary to the abovementioned post-graduate program, this project focuses on malleable robotic systems instead of formable tissue. The participating scientific institutions are distributed all over Germany and do not work on explicitly medical fields. However, some themes like continuum robotics may become interesting for medical applications in the course of the funding period.

## 4. Conclusions

RAS is already regularly used in specialized centers for head and neck surgery. In simple surgeries and anatomical easily reachable areas like the oropharynx, a distinct advantage of the RAS remains to be proved in randomized clinical trials. Especially, a comparison to current surgical standard procedures is inevitable. For some medical indications like the neck dissection, the established open surgical access will certainly not be replaced by the RAS. This is among other things due to the increased required time and the financial additional effort. Potential for routine use can be seen for surgery of the skull base, the nasopharynx, or the larynx in patients with difficult anatomical features. For this purpose, systems with surgical instruments designed especially for the field of head and neck surgery would have to be developed. The aim must be to apply the new technology reasonable and advantageous for patient care and to analyze the information gained from clinical trials attentively. Despite the current obstacles, we strongly believe that robotic systems will have a major influence on the way how we will perform surgery in the head and neck region during the next decade. Robotic systems will help the surgeon to perform better surgery in difficult-to-reach areas, to reduce surgical morbidity, and to increase patient safety.

## Figures and Tables

**Table 1:** Summary of commercially available robotic systems with FDA/CE approval and possible application in head and neck surgery.

**DaVinci Xi^®^** **Multi-Port Robotic System**	**DaVinci Single-Port (SP)^®^** **Single-Port Robotic System**	**Flex^®^ Robotic System** **Flexible Single-Port Robotic System**	**Versius^®^, Senhance^®^ Surgical System** **Multi-Port Robotic Systems**
**Fields of application**			
Oral cavity/oropharynx Supraglottis Thyroid gland through retroauricular or transaxillary access	Oral cavity Oropharynx Supraglottis Glottis and hypopharynx questionable	Oropharynx Hypopharynx Larynx (supraglottis, glottis)	Abdominal surgery
**Advantages**			
+ Most established system for TORS + Good availability in many surgical centers + Good access to oral cavity and tongue base with the aid of retractors	+ 3 instruments and the camera can be used simultaneously in a small space + Flexible control of instruments and camera	+ Developed especially for the head and neck area + Good adaptation to the pharynx and narrow anatomical regions + No need for head reclination, + small mouth opening sufficient	+ easy access due to flexible positioning of the robotic working ports (both) + System provides haptic-feedback and + standard re-usable instruments for lowering costs (Senhance)
**Disadvantages**			
- Frequent necessity of large retractors, like the Feyh-Kastenbauer-retractor with associated morbidity - Large instruments initially developed for abdominal and genitourinary surgery - Rigid instruments that can injure the pharynx/teeth/jaw upon navigating, during collision with the retractor due to high leverage and the missing of a haptic feedback	- Instruments originally developed for abdominal and genitourinary surgery - Instruments’ dimensions too large for microsurgery of the larynx - Single-port is rigid. Reclination of the head is required	- Time-consuming and difficult system positioning - Often necessity of manual position correction - No broad availability on the market yet	- Currently only CE certification (Versius) - FDA and CE approval currently only for abdominal surgery (Senhance) - Application only in a few hospitals and only in abdominal surgery so far - No distinct surgical instruments for head and neck surgery, no adaptations on the narrow head and neck surgical fields
**RoboticScope^®^** **Robotic Exoscope**	**VITOM^®^ 3D HD** **Manual or Robotic Exoscope**	**ORBEYE^®^** **Manual Exoscope with Multispectral Imaging**	**Cirq^®^** **Robot-Assisted Endoscope Guidance System**
**Fields of application**			
Pharynx/oral cavity Microscopic vessel anastomosis in reconstructive surgery Otologic surgery	Pharynx/oral cavity Otologic surgery Surgical procedures of the neck	Neurosurgery Surgical procedures of the neck Microvascular anastomosis	Paranasal sinus surgery Nasolacrimal duct Spine surgery
**Advantages**			
+ Free handed bimanual instrumentation + Camera view independent from head and body position + Ergonomic working position 3D head-mounted display	+ 3D view on a single 4K HD monitor + 3D view on the monitor with special glasses available for the whole surgical team + Ergonomic working position	+ Conventional white-light imaging in 3D with 4K-resolution + Fluorescence imaging modes + Combination with modern narrow-band imaging (NBI)	+ Bimanual instrumentation + System positioning entirely via foot pedal + Compatible with any standard size endoscope + Very stable visualization of the surgical field due to missing of natural tremor
**Disadvantages**			
- Unusual visualization as the visual line does not necessarily point towards the surgical site - Likely longer setup time than conventional operating microscope - No application in narrow anatomical areas	- Controlling via mechanical holder correlates with a more difficult repositioning in comparison to a conventional operating microscope - Controlling via robotic arm requires interruption of the surgery for repositioning with one hand removed from the surgical field	- Motorized repositioning of the camera via foot pedal is only possible in x- and y-axis - Fine adaptions require manual repositioning or rotations - Using external monitors requires a cognitive adjustment	- No irrigation system removal of the system for cleaning when fogging and staining

**Table 2:** Government initiatives for general Robotic Research & Development worldwide.

Title	Country/Time	Budget in Million USD
Development Plan of the Robot Industry	China 2016–2020	577 total
Key Special Program on Intelligent Robots	China 2019	577 total
New Robot Strategy	Japan 2016–2020	351 total (53.6 *)
Implementation Plan for Intelligent Robots	Korea 2018	150 total (0.84 *)
The 3rd Basic Plan on Intelligent Robots	Korea 2019–2023	126 for 2020
Horizon 2020 ICT Robotics Work Program	EU 2014–2020	780 total (5 *)
National Robotics Initiative 2.0	The United States since 2016	35 for 2019

* funding especially for healthcare and medical robotics.

**Table 3:** Research initiatives with a focus on Robotics.

Title	Form of Research Promotion	Speaker and Web Page
SARAS project	EU-Promotion Horizon 202 (project 779813)	Riccardo Muradore, Verona, Italy www.saras-project.eu †
Robotics Technology Development and Deployment	National Institutes of Health * (Funding No. PAR-10-279) since 2011	www.grants.nih.gov/grants/guide/pa-files/PAR-10-279.html †
Development of Single Port Surgical Robot for Flexible Joints for Light Oral or Laparoscopic Surgery	Ministry of Trade, Industry and Energy, Korea 2018	www.motie.go.kr/www/main.do †
SMARTsurg	EU-Promotion Horizon 2020 (project 732515) since 2017	Sanja Dogramadzi, Bristol, UK www.smartsurg-project.eu †
Soft tissue robotics	DFG post-graduate program (GRK 2198) since 2017	Oliver Röhrle, Stuttgart www.str.uni-stuttgart.de †
Soft material robotics	DFG priority program (SPP 2100) since 2019	Annika Raatz, Hannover www.spp2100.de †

* funding especially for healthcare and medical robotics, † last accessed on 31 January 2021.
